# Pueperal Insanity

**Published:** 1876

**Authors:** L. R. Landfear

**Affiliations:** Dayton, Ohio


### "PUEPERAL INSANITY."

Read before the "Society of Alumni of Miami Medical College," September 23 1875. By L. R. Landfear, M. D., Dayton, O.

*Mr. President and Gentlemen:*

While looking around for a subject which would be of general interest to the members of this Association, my attention has been directed to a class of cases, to be met with in the practice of nearly every physician, and which "the" nothing new may be adduced for those who have spent long years in the practice of medicine, cannot but have *some* interest for those who, like myself, are comparatively young in the profession.

I wish, therefore, to call your attention briefly to the subject of "Puerperal Insanity." To be able to detect the first low mutterings of the coming storm; the insomnia, malaise, irritability, restlessness and unevenness of spirits; the pains in the head, the white and coated tongue, the constipated bowels; the quick and weak pulse; the melancholy distrust, suspicion or perhaps some slight hallucination in regard to some one thing, each and all promising to develop into an attack of Melancholia or Acute Mania so fraught with anxiety to friends; with care and vexation to the Practitioner; to be able, I say, to detect all these symptoms and read them aright, is, in many cases, to avert the threatened danger. Immediate and active treatment is imperatively demanded, which, if neglected, may lead to the most lamentable results.

It has been customary to designate all cases, occurring between the beginning of pregnancy and the close of lactition, as Puerperal Insanity; more recently it has been subdivided into the Insanity of Pregnancy, of the Puerperal state, and of Lactition; "though" all are still included under the general head of Puerperal Insanity. The delirium, often occurring during labor, by some authors, is claimed to be still another division; but it is not generally recognized.

Cases of insanity during pregnancy are far less frequent than those occurring afterward. The periods of utero-gestation at which we may look for the majority of cases, are, according to Dr. Tuke, those which are generally considered critical, viz: the third, fifth and seventh months. The majority of cases terminate with the occurrence of labor.

Between the two following divisions the line of demarkation is not so clearly drawn. By some it is claimed that the period of true puerperal insanity is during the first two weeks after labor; while others give a much longer time. The majority of cases, however, occur during the first two months after delivery.

Following this form, is the insanity caused by undue or protracted lactition. The injudicious and persistent nursing of infants, when there is an evident impairment of strength, as evinced by loss of appetite, weak pulse, languor and anemia, is a fruitful source of insanity.

Dr. Forbes Winslow has called attention to what he characterizes as a "stage of Incubation," during which, as has been before intimated, we may hope for the most beneficial results from treatment. In an asylum practice this stage is rarely met with. It is only after weeks, and perhaps months, of incessant care and watching that the friends, worn out and discouraged, it may be, consent to the removal of the patient to an asylum. Before that point is reached the milder cases recover.

Melancholia and Mania are the two forms met with, differing in no respects as regards symptoms and character from the insanity resulting from other causes.

It has been claimed by some that the profanity and vulgarity of puerperal patients is so marked as to distinguish them from all others, an opinion controverted by the best authorities, by observation and statistics.

The suicidal impulse is *very* frequently present, as a source of unceasing care and anxiety to those having them in charge. Especially is this the case in the melancholia of pregnancy and the lactition, where the persistence and cunning displayed in eluding the vigilance of nurses is often wonderful.

Much has been said and written, and much speculation indulged in, as to the cause of "Puerperal Insanity," and yet the matter is in doubt. Pathological research throws but little light on the subject. Where leisions are found on post mortem examination, they differ in no way from those found in the insane from other causes. One fact, however, is well authenticated, that in a very large number of cases it is dependent upon, or associated with, an hereditary predisposition, and could the complete genealogy of every patient be ascertained, the number would be found to be far greater than is generally supposed.

As to the exciting causes there is also a diversity of opinion. The earlier writers supposed that all puerperal diseases were caused by suppressed secretions or a metastasis of milk to the brain; some even claiming to have discovered milk within the cranium—a theory which finds few advocates at the present day. More recently the hypothesis has been advanced that the cause may be found in the excited condition of the whole sexual system during the puerperal period. The intimate relation existing between the brain and sexual system at other times, often being sufficient to produce an attack of insanity when the functions are interferred with, serve as an argument for those who advocate this doctrine, and the nervous susceptibility of the lying-in woman being so much greater and so common is claimed as a good ground for her belief.

The Anæmic and exhausted condition of many patients suffering from puerperal insanity has led some to suppose that the cause may be found in the Anæmia and exhaustion consequent upon the puerperal condition; but this hypothesis is applicable to comparatively few cases. Possibly it may be in some cases a predisposing but scarcely an exciting cause.

Sir James Y. Simpson, however, claims that it is due to a state of blood poisoning or toxemia—basing his belief on the similarity of the phenomena, at their supervention, to the blood poisoning of alcohol and the narcotic poisons. But "to what morbid change or to what special morbid agent in the blood it is due," he candidly confesses that he does not know. As an argument in favor of his view, he states that "in a large proportion of cases there is present, at the commencement, a marked state of Albumenuria." Of the ten cases observed by him, in eight albumen was found in the urine. He further observes, that "where albumenuria exists there is always a diminished elimination of urea and other excrementicious material which usually pass off with the urine. Urea accumulated in morbid excess in the circulating system does not *per se*, or as urea, produce any special poisonous effects; but to the sudden excess of carbonate of ammonia in the circulation, a salt which urea readily forms when it becomes decomposed, is due to Albumenuria resulting in convulsions, coma, etc. That when the whole chemistry of the blood becomes deranged, as is always liable to happen in Puerperal Albumenuria, other toxicological agents may become developed, possibly of an alkaloidial character, which may be as certain of exciting delirium and insanity as an overdose of morphia or other poisonous vegetable alkaloid is certain of producing their special toxicological effects. In the blood of the puerperal female, modified as it is in pregnancy and delivery, and containing the effete elements of the disintegrating uterus and the materials for the new lacteal secretions, ferments and agents may possibly exist which are more apt to develop special morbid poisons out of the retained venal excretions than happens in other states of the system." This, in brief, is the above named author's view, and he concludes by saying that "the whole subject is yet quite dark and conjectural, and will remain so until pathological chemistry is able to cast some light upon it." In an asylum practice it is extremely rare, if ever, that Albumen is found in the urine of a puerperal patient, as it is seldom found except at the very onset of the attack, and is of but one or two days duration: frequently of but a few hours.

Another exciting cause is due to mental emotions. Often a shock, which, under other circumstances, would produce no ill effect whatever, in a puerperal patient may be the feather which turns the scale and precipitates the patient into a condition of the most violent insanity. For this reason it is claimed that single females are much more liable to suffer from this malady than the married, on account of the depressed condition of the mind during pregnancy and the puerperal state caused by the unfortunate position. In some of the French hospitals, statistics show that a very large per cent. of the puerperal patients are single; greater by far than can be found in the hospitals of Great Britain or the United States. It may also be added that the disease is of more frequent occurrence in first than in subsequent labors, and in unnatural and tedious than in natural ones.

The progress is very favorable. Even in an asylum, where only the more serious, and what might be called the chronic, cases are treated, the majority recover and comparatively few die. But from the greater number treated outside, by the general Practitioner, it is impossible to secure statistics which are at all reliable.

From the Records of the "Western Ohio Hospital for the Insane," I have collected a few statistics which may be of interest in connection with this subject. Unfortunately, on some important points the Records are silent.

The whole number of females treated from September 1, 1855, to November 15, 1874, was seventeen hundred and seventy-five (1775), of which number two hundred and five (205) were marked "Puerperal," divided as follows: Pregnancy, twenty-eight (28), Puerperal State, one hundred and fifty-eight (158), Lactition, nineteen (19). One hundred and twenty-three (123) were discharged, Recovered, "eleven" (11), Improved, twenty-nine (29), Unimproved, thirteen (13), died, and twenty-nine (29) were still remaining November 15, 1874. In one hundred and fifty-five (155) cases it was the first attack, in thirty (30) the second, in eight (8) the third, in two (2) the fourth, in one (1) the fifth, and in nine (9) unknown. Two hundred and one (201) were married, and but four (4) single. From the above it will be seen that more than 55 per cent. were discharged as "Recovered," while undoubtedly nearly, if not all, who were removed by friends, as improved, eventually recovered. The average duration of attack, before admission in those who recovered, was 3 months and 27 days, and the averuge duration of residence in hospital was 5 months and 15 days. Average duration before admission in the improved 1 year, 6 months and 25 days; in the unimproved, 2 years, 2 months and 17 days. Of those remaining at the close of the year several have since gone to their homes cured. Of the thirteen (13) deaths, three (3) only were from the exhaustion of Acute Mania. All others were from the intercurrent affections, such as Phthisis, Marasmus, Epilepsy, etc., the average duration of residence being 3 years, 7 months and 10 days.

While the majority of cases recover, there are always two dangers to be dreaded—the danger to life and reason. With Acute Mania, the rapid and irritable pulse, running up to 120 or 130 per minute, is seldom unattended with danger to the patient's life, not unfrequently, however, even under these unfavorable circumstances, the patients do recover, and that very rapidly.

At other times the bodily powers are restored while the mind is lost in an almost hopeless dementia. The principal danger to life is from exhaustion, and it is indeed wonderful that so many recover from the effects of such intense excitement, fatigue and loss of sleep.

In the treatment of Puerperal Insanity, as in that of so many other diseases, a remarkable revolution has taken place; and, with the adoption of a more rational treatment, a corresponding decrease of the rate of mortality. Formerly venesection was resorted to on every occasion. No matter what the condition, or how great the anæmia, the patient must be bled. The cold shower bath, and occasionally the surprise bath, were called into requisition; the latter consisting of a dark room with a trap in the floor, through which the patient, in her wandering about the room, was precipitated up to her arm-pits in a cold bath.

All this has now been done away with, and the attention of the Physician is directed to the building-up rather than the pulling-down process. Especially is this the case with the anæmic and exhausted patient, prostrated from loss of blood, protracted lactition or insomnia. In such cases tonics and calybiates, in addition to a nourishing diet, are imperatively demanded. No preparation of iron is, perhaps, better suited to this class of cases than the Tincture of Perchloride, assisting, as it does, in the recovery of patients who have become exsanguine from loss of blood following or during labor or from prolonged lactition.

In the early stages, purgation is recommended by the best authorities. A brisk purgative, bringing away large and offensive stools, has been known, in numerous cases, to restore the patient to reason. Beneficial results often also follow the administration of an emetic where there is evidently an overloaded stomach with foul and coated tongue.

In a certain class of cases, where there is great excitement, and depletion would seem to be indicated, excellent results will frequently be obtained from the use of vascular and nervous sedatives. Warm baths, also, exert a most soothing influence.

The various anodynes, with a proper discrimination, play a most important part in the treatment. The various preparations of opium are perhaps most frequently employed, but, in order that it may produce the most satisfactory results, it must be given in large doses, small doses often having a tendency to increase rather than allay the irritation. In very many cases opium seems of no avail, and recourse must be had to other remedies. Hydrâte of Chloral, Conium, Hyosciamus, Canabis Indica, and many others, are all, in turn, valuable agents. In Bromide of Potassum we have another valuable remedy, which often produces a peaceful sleep after all other means have failed.

In connection with the physical there is also a moral treatment which should by no means be neglected. Change of scene, separation from sympathising but injudicious friends, a removal, so far as may be, from old, and possibly disturbing associations; the presence of entire strangers and the care of experienced nurses, with firm yet kindly supervision, are of incalculable benefit to patients suffering from this as well as from other forms of insanity. The speedy recovery of many a puerperal patient when removed to an asylum, is, I believe, due quite as much to these causes as to the physical treatment received.

From these remarks I would not be understood as advocating the immediate removal to an asylum of all patients suffering from puerperal insanity. Many, and perhaps the majority, may be successfully treated at home by the intelligent physician. Indeed the practice of some physicians of "shipping off" these troublesome patients to an asylum, without regard to their physical condition, can not be too strongly deprecated. Such cases are not so infrequent as might be supposed, and reflect but little credit upon the heart or intelligence of the attending physician.

In conclusion, gentlemen, I cannot but express to you how fully I realize the imperfections of this paper, and how imperfectly the subject has been brought before you; but, if I have succeeded in throwing a single ray of light upon the subject, for those who have never given it particular attention, I shall feel that my time has not been wholly misspent, and my object in writing it will have been accomplished.

